# HSP70 attenuates compression-induced apoptosis of nucleus pulposus cells by suppressing mitochondrial fission via upregulating the expression of SIRT3

**DOI:** 10.1038/s12276-022-00745-9

**Published:** 2022-03-25

**Authors:** Binwu Hu, Peng Wang, Shuo Zhang, Weijian Liu, Xiao Lv, Deyao Shi, Lei Zhao, Hongjian Liu, Baichuan Wang, Songfeng Chen, Zengwu Shao

**Affiliations:** 1 Department of Orthopaedics, Union Hospital, Tongji Medical College, Huazhong University of Science and Technology, Wuhan, 430022 China; 2 Department of Orthopaedic Surgery, The First Affiliated Hospital of Zhengzhou University, Zhengzhou, China

**Keywords:** Rheumatic diseases, Apoptosis

## Abstract

Compression-induced apoptosis of nucleus pulposus (NP) cells plays a pivotal role in the pathogenesis of intervertebral disc degeneration (IVDD). Recent studies have shown that the dysregulation of mitochondrial fission and fusion is implicated in the pathogenesis of a variety of diseases. However, its role in and regulatory effects on compression-induced apoptosis of NP cells have not yet been fully elucidated. Heat shock protein 70 (HSP70) is a major cytoprotective heat shock protein, but its physiological role in IVDD, especially its effect on mitochondrial fission and fusion, is still unknown. Herein, we found that compression could induce mitochondrial fission, which ultimately trigger apoptosis of NP cells via the mitochondrial apoptotic pathway. In addition, we identified the cytoprotective effects of HSP70 on NP cells, and we found that promoting the expression of HSP70 could protect NP cells from abnormal mechanical loading in vitro and in vivo. Finally, we showed that HSP70 inhibited compression-induced mitochondrial fission by promoting SIRT3 expression, thereby attenuating mitochondrial dysfunction and the production of reactive oxygen species and ultimately inhibiting the mitochondrial apoptotic pathway in NP cells. In conclusion, our results demonstrated that HSP70 could attenuate compression-induced apoptosis of NP cells by suppressing mitochondrial fission via upregulating SIRT3 expression. Promoting the expression of HSP70 might be a novel strategy for the treatment of IVDD.

## Intervertebral disc degeneration: Protein protects against compression-induced cell death

A so-called chaperone protein that assists other proteins in correctly folding helps to prevent compression-induced cell death in the intervertebral discs responsible for cushioning the spine. Binwu Hu from Huazhong University of Science and Technology, Wuhan, China, and coworkers showed that mitochondria in the cells from the jelly-like substance found in vertebral discs in the spine tended to divide when exposed to abnormal mechanical loading. This fission resulted in cell death. In cell culture experiments and in mice, the researchers found that boosting levels of the molecular chaperone HSP70 (heat shock protein 70) prevented this mitochondrial dysfunction, in part by activating another stress-response protein called SIRT3. The findings point to HSP70 as a promising drug target for addressing intervertebral disc degeneration, a common cause of chronic back pain.

## Introduction

Intervertebral disc degeneration (IVDD) is widely recognized as the pathological basis of multiple spinal disorders, such as disc herniation, low back pain, and spinal canal stenosis, resulting in heavy social and economic burdens^1,2^. Although the precise mechanisms have not been fully elucidated, nucleus pulposus (NP) cell apoptosis has been proven to be critical in the pathogenesis of IVDD^3^. The prominent function of the IVD is to transfer load, dissipate energy and facilitate joint mobility, which subjects NP cells to mechanical loading insults^4^. Previous studies have demonstrated that high-magnitude compressive loading could trigger apoptosis of NP cells, leading to the development of IVDD^5^. However, the mechanisms of compression-induced apoptosis of NP cells and their regulatory strategies remain to be elucidated.

Mitochondria are crucial cell death executioners whose dysfunction plays a pivotal role in compression-induced apoptosis of NP cells^5,6^. Mitochondria are highly dynamic organelles that continuously undergo fission and fusion^7^. The regulation of mitochondrial fission and fusion in mammalian cells involves a large number of molecules, including the fusion proteins mitofusin 1/2 (Mfn1/2) and optic atrophy 1 (OPA1) and the fission proteins dynamin-related protein 1 (DRP1), mitochondrial fission factor (MFF), and mitochondrial fission 1 protein (Fis1)^7^. In response to stressors, fission and fusion are important for maintaining functional mitochondria. Fusion can promote complementation between damaged mitochondria, while fission contributes to the removal of damaged mitochondria^8,9^. Disruption of the dynamic balance between mitochondrial fission and fusion damages normal mitochondrial and cellular functions, ultimately leading to cell apoptosis^7,10^. An imbalance in mitochondrial fission and fusion has been implicated in the pathogenesis and progression of a variety of diseases, such as tumors, cardiovascular diseases, diabetes and osteoarthritis^11,12^. Regarding IVDD, previous studies have demonstrated that compression treatment could promote the expression of mitochondrial fission-related proteins and downregulate the expression of mitochondrial fusion-related proteins^13,14^. In addition, inhibiting DRP1 was proven to relieve compression-induced mitochondrial dysfunction and apoptosis of NP cells^14^. These results indicate that compression might trigger apoptosis of NP cells by inducing mitochondrial fission. However, the causal relationship between compression-induced mitochondrial fission and apoptosis of NP cells also needs further validation.

Silent information regulator 2 proteins (sirtuins) are highly conserved NAD^+^-dependent protein deacetylases that participate in multiple pathophysiological processes^15^. SIRT3 is the most widely studied sirtuin family member, which is mainly located in mitochondria and is extensively involved in the regulation of energy metabolism, oxidative stress, inflammation, apoptosis and autophagy^16,17^. Increasing evidence has established that SIRT3 is involved in the regulation of mitochondrial fission and fusion. SIRT3 could suppress mitochondrial fission by inhibiting the expression of Fis1 and promoting the expression and activation of OPA1^18–20^. Moreover, SIRT3 was proven to inhibit mitochondrial fission induced by oxidative stress in NP cells^21^. However, whether SIRT3 regulates the compression-induced imbalance of mitochondrial fission and fusion in NP cells remains unclear.

Heat shock protein 70 (HSP70) is a major cytoprotective HSP that assists the folding, transport and assembly of proteins and controls the activity of regulatory proteins^22^. Increased expression of HSP70 has been detected in various diseases and pathological processes, and the induction of HSP70 could efficiently slow the progression of many diseases^23,24^. The expression of HSP70 was also shown to be elevated in degenerated IVDs^25^. Moreover, several in vitro studies revealed that adverse microenvironmental factors such as hypoxia, hypertonia and compression could upregulate the expression of HSP70 in NP cells^26–28^. However, the specific roles of HSP70 in the pathogenesis and progression of IVDD remain unclear.

Numerous studies have demonstrated that HSP70 can suppress mitochondrial dysfunction, but whether HSP70 achieves this effect by regulating mitochondrial fission and fusion has not been reported. A previous study showed that the loss of mitochondrial HSP70 function makes the tubular network of mitochondria collapse to form aggregates in yeast cells, indicating the potential of HSP70 to regulate mitochondrial fission and fusion^29^. In addition, it was proven that both SIRT1 and SIRT3 could interact with HSP70, and HSP70 has been demonstrated to regulate the expression of SIRT1^30,31^. Therefore, it is reasonable to hypothesize that HSP70 might also regulate SIRT3 expression. Taking all of the evidence into account, we hypothesize that HSP70 attenuates compression-induced apoptosis of NP cells by controlling the balance of mitochondrial fission and fusion via regulating SIRT3 expression.

## Materials and methods

### Collection of human NP samples

Experimental protocols for the present study were approved by the Medical Ethics Committee of Tongji Medical College, Huazhong University of Science and Technology. Written informed consent was obtained from all patients. Human NP tissues were obtained from patients undergoing routine lumbar surgery due to lumbar idiopathic scoliosis, lumbar burst fracture, spinal stenosis, and lumbar disc herniation. The degree of IVDD was assessed according to the Pfirrmann magnetic resonance imaging grading system, and samples with Pfirrmann grades I and II were regarded as nondegenerated^32^.

### Isolation and culture of rat NP cells

All animal procedures in the present study were conducted under the approval of the Animal Experimentation Committee of Huazhong University of Science and Technology. NP cells were extracted from rat tail NP tissues as described previously^6,33^.

### Cell treatments

A pressure vessel was used to simulate the in vivo compressive environment according to protocols previously established by our group^6,33^. The DRP1 inhibitor Mdivi-1 (Selleck Chemicals, Houston, TX, USA) was administered alone or in combination with the mitochondrial fission activator FCCP (Selleck Chemicals) 1 h before compression treatment. To induce the expression of HSP70, NP cells were pretreated with TRC051384 (TRC, Selleck Chemicals) for 24 h. The HSP70 inhibitor Ver155008 (VER, Selleck Chemicals), SIRT3 inhibitor 3-TYP (Selleck Chemicals), or FCCP was administered in combination with TRC 24 h before compression treatment.

### Transmission electron microscopy (TEM)

Following treatment, NP cells were collected by trypsinization and washed twice with PBS. Then, the cell pellets were fixed with 2.5% glutaraldehyde in PBS for 2 h and postfixed with 1% osmium tetroxide for 2 h at room temperature. Next, the pellets were dehydrated and embedded in epon 812. Ultrathin sections were stained with uranyl acetate and lead citrate and examined by TEM (HT7700, Hitachi, Tokyo, Japan).

### Live and dead cell staining

For live and dead cell staining, NP cells were seeded in 12-well culture plates. After the indicated treatments, the cells were gently washed twice with PBS and incubated with calcein-AM (Santa Cruz Biotechnology, Inc., Dallas, TX, USA) at 37 °C for 20 min in the dark. Then, the cells were further stained with propidium iodide (PI, Nanjing Keygen Biotech, Nanjing, China) according to the manufacturer’s instructions. Fluorescence microscopy (Olympus IX71, Olympus Inc., Tokyo, Japan) was used to observe and photograph the live (green fluorescence) and dead (red fluorescence) cells.

### Terminal deoxynucleotidyl transferase biotin-dUTP nick end labeling (TUNEL) assay

TUNEL assays were performed using the One Step TUNEL Apoptosis Assay Kit (Beyotime, Shanghai, China). Briefly, after the indicated treatments, NP cells seeded on glass coverslips were fixed with 4% paraformaldehyde for 30 min and permeabilized with 0.3% Triton X-100 for 5 min at room temperature. Then, the cells were incubated with the TUNEL reaction mixture for 1 h at 37 °C in the dark and counterstained with 4′-6-diamidino-2-phenylindole (DAPI). Cells with red fluorescence were TUNEL-positive, and images were obtained using a fluorescence microscope (Olympus BX53, Olympus Inc., Tokyo, Japan).

### Detection of mitochondrial reactive oxygen species (mtROS) and cellular ROS

The mtROS and cellular ROS were analyzed using MitoSOX Red (Thermo Fisher Scientific, Waltham, MA, USA) and 2′-7′-dihydrodichlorofluoroscein diacetate (DCFH-DA, Beyotime), respectively, according to the manufacturer’s instructions. After the indicated treatments, NP cells were harvested by trypsinization, washed twice with PBS, and incubated with MitoSOX Red for 10 min or DCFH-DA for 20 min at 37 °C in the dark. Then, the cells were washed with PBS three times, and the fluorescence intensity was measured using flow cytometry (BD LSR II).

### Measurement of cellular ATP

Cellular ATP levels were assessed using an Enhanced ATP Assay Kit (Beyotime). Briefly, NP cells were lysed with ATP lysis buffer. Then, the lysate was centrifuged at 12,000 × *g* for 5 min, and the supernatant was reacted with the ATP detection working solution. Luminescence activity was measured by a VICTOR Nivo Multimode Reader (PerkinElmer), and the ATP level was normalized to the cellular protein concentration.

### Transfection of siRNA

siRNAs against HSP70 were synthesized by RiboBio Co. (Guangzhou, China). The target sequences of the siRNAs were as follows: *HSPA1A*, 5′-GTAAAGAAGTCTTCAGCAA-3′; and *HSPA1B*, 5′-GCGAGGCTGACAAGAAGAA-3′. The negative control (NC) siRNA was also synthesized by RiboBio Co. Transfection was performed using Lipofectamine 3000 reagent (Thermo Fisher Scientific) according to the manufacturer’s instructions. Transfection efficiency was measured by Western blotting after 48 h of transfection.

### Immunofluorescence (IF) staining

Following the treatments, NP cells seeded on glass coverslips were gently washed twice with PBS and fixed with 4% paraformaldehyde for 15 min. The cells were then permeabilized with 0.5% Triton X-100 for 20 min and blocked with goat serum albumin for 1 h at room temperature. Samples were subsequently incubated with primary antibodies against TOM20 (1:200, Cell Signaling Technology, Danvers, MA), matrix metalloproteinase 3 (1:200, Proteintech, Wuhan, China), matrix metalloproteinase 13 (1:200, Proteintech), and SIRT3 (1:200, ABclonal, Wuhan, China) at 4 °C overnight. After being washed with PBST three times, the samples were incubated with fluorophore-conjugated secondary antibodies (1:200, Servicebio, Wuhan, China) for 1 h. The nuclei were stained with DAPI. Fluorescence images were observed using a fluorescence microscope (Olympus BX53).

### Western blot analysis

After the indicated treatments, NP cells were washed twice with PBS and lysed in radioimmunoprecipitation assay (RIPA) lysis buffer (Beyotime) supplemented with phosphatase and protease inhibitors. The lysates were electrophoresed by 10–12% sodium dodecyl sulfate–polyacrylamide gel electrophoresis (SDS–PAGE) and transferred onto polyvinylidene difluoride (PVDF) membranes (EMD Millipore, Billerica, MA, USA). Then, the membranes were blocked with NcmBlot blocking buffer (NCM Biotech, Jiangsu, China) for 20 min at room temperature and incubated overnight at 4 °C with primary antibodies against HSP70 (1:1000, ABclonal), Mfn1 (1:1500, Proteintech), Mfn2 (1:1500, Proteintech), OPA1 (1:1000, Proteintech), DRP1 (1:1000, Affinity Biosciences), MFF (1:1000, Proteintech), Fis1 (1:1000, Proteintech), cleaved caspase 3 (1:1000, Affinity Biosciences, OH, USA), caspase 9 (1:1000, Proteintech), Bcl-2 (1:1000, Abcam, Cambridge, MA, USA), Bax (1:1000, Proteintech), Collagen II (1:500, Affinity Biosciences), Aggrecan (1:500, NOVUS, USA), SIRT3 (1:1000, ABclonal), and β-actin (1:2000, Cell Signaling Technology, Danvers, MA, USA). Then, the PVDF membranes were washed with TBST and incubated with horseradish peroxidase-conjugated secondary antibodies for 1 h at room temperature. Signals were detected using an ECL kit (Affinity Biosciences).

### Quantitative real-time PCR (qRT-PCR) analysis

Total RNA was extracted from NP cells using RNA-easy isolation reagent (Vazyme Biotech Co., Ltd, Nanjing, China). Then, the concentration and purity of the total RNA were evaluated by a Nanodrop 2000. First strand cDNA was synthesized using a cDNA synthesis kit (Yeasen, Shanghai, China). qRT-PCR was performed using SYBR Green Master Mix (Yeasen) on a CFX Connect Real-Time PCR System (Bio-Rad, Hercules, CA, USA). The relative expression of target genes was normalized to *GAPDH* and calculated using the 2^−ΔΔCt^ method. The primer sequences were as follows: *HSPA1A*, forward, 5′-TCCGTGTGTATGTTGGGAGG-3′, reverse, 5′-AACCCCATTTCCCCGTCATC-3′; *HSPA1B*, forward, 5′-AGGTCTTGTCTGCCTCCGATTT-3′, reverse, 5′-GGAAGCCACCATCCTTAGGTC-3′; and *GAPDH*, forward, 5′-ACAGCAACAGGGTGGTGGAC-3′, reverse, 5′-TTTGAGGGTGCAGCGAACTT-3′.

### Animal surgery

Thirty skeletally mature 3-month-old male C57BL/6J mice were purchased from the Experimental Animal Center of Tongji Medical College, Huazhong University of Science and Technology, China. The mice were randomly divided into a sham-operated group, lumbar spine instability (LSI) group and LSI-TRC group. For the LSI operation, the mice were anesthetized by an intraperitoneal injection of 50 mg/kg pentobarbital. Then, the 3rd-5th lumbar spinous processes, as well as the supraspinous and interspinous ligaments, were resected to cause instability in the lumbar spine. The sham-operated group only underwent a skin incision. One week after the operation, mice in the LSI-TRC group were intraperitoneally injected with 4.5 mg/kg TRC, while the mice in the sham-operated and LSI groups were intraperitoneally injected with an equivalent volume of vehicle (dimethyl sulfoxide) every 2 days. The mice were euthanized 8 weeks after the operation, and spine samples were harvested, fixed in 4% paraformaldehyde, decalcified in 10% ethylenediaminetetraacetic acid and embedded in paraffin.

### Immunohistochemical (IHC) staining

IHC staining was performed as previously described^34^. The primary antibodies used in the present study were as follows: Mfn1 (1:500, Proteintech), Mfn2 (1:200, Proteintech), DRP1 (1:200, Affinity Biosciences), MFF (1:300, Proteintech), HSP70 (1:400 for human NP tissue and 1:100 for mice NP tissue, ABclonal), matrix metalloproteinase 13 (1:250, Proteintech), cleaved caspase 3 (1:120, Affinity Biosciences), and SIRT3 (1:50, ABclonal). The integrated optical density (IOD) of the IHC images was quantified using NIH ImageJ analysis software (ImageJ) according to previously established procedures^35^.

### Statistical analysis

The data are presented as the mean ± standard deviation of at least three independent experiments. All statistical analyses were performed using GraphPad Prism V.8.0 software (GraphPad Software, San Diego, CA, USA). Differences between groups were analyzed by Student’s *t* test or one-way analysis of variance followed by the least significant difference test. *P* < 0.05 was considered statistically significant.

## Results

### Compression induced mitochondrial fission in NP cells

We first measured the expression levels of mitochondrial fission- and fusion-related proteins in normal and degenerated human NP tissues. IHC showed that the expression levels of DRP1 and MFF were increased in degenerated NP tissues, while the expression levels of Mfn1 and Mfn2 were decreased (Supplementary Fig. 1a). Then, we examined the effects of compression on mitochondrial fission and fusion in NP cells. The Western blot results showed that compression increased the expression of DRP1, MFF and Fis1 and downregulated the expression of Mfn1, Mfn2, and OPA1 (Supplementary Fig. 1b). IF staining of TOM20 demonstrated that the lengths of mitochondria in NP cells gradually decreased in a time-dependent manner (Supplementary Fig. 1c). In addition, we also performed a TEM assay to observe mitochondrial ultrastructure. TEM showed that compression decreased the lengths of mitochondria in NP cells (Supplementary Fig. 1d). Taken together, these results proved that compression could induce mitochondrial fission in NP cells.

### Inhibiting mitochondrial fission attenuated compression-induced apoptosis of NP cells

To clarify the role of compression-induced mitochondrial fission in apoptosis of NP cells, the DRP1 inhibitor Mdivi-1 was used. The results of the Cell Counting Kit-8 (CCK-8) assays revealed that Mdivi-1 treatment attenuated the loss of viability caused by compression (Supplementary Fig. 2a). IF staining showed that Mdivi-1 (20 μM) treatment efficiently suppressed the fission of mitochondria in NP cells (Supplementary Fig. 2b). We then determined the effect of Mdivi-1 treatment on apoptosis of NP cells. As expected, flow cytometric analyses demonstrated that inhibiting mitochondrial fission alleviated compression-induced apoptosis of NP cells (Supplementary Fig. 2c, d). To determine whether the cytoprotective effects of Mdivi-1 were due to the suppression of mitochondrial fission, the mitochondrial fission activator FCCP was used. Notably, FCCP treatment impaired the preventive effects of Mdivi-1 on the loss of viability in NP cells in response to compression (Supplementary Fig. 2e). IF staining showed that FCCP (5 μM) markedly recalled mitochondrial fission in NP cells (Supplementary Fig. 2f). Furthermore, FCCP treatment also abolished the inhibitory effects of Mdivi-1 on compression-induced apoptosis of NP cells (Supplementary Fig. 2g, h). Collectively, these data suggested that compression-induced mitochondrial fission could trigger apoptosis of NP cells.

### Inhibiting mitochondrial fission suppressed the mitochondrial apoptotic pathway

To explain the mechanism of the proapoptotic effects of mitochondrial fission, we focused on the mitochondrial apoptotic pathway. We first measured mitochondrial function in NP cells. Flow cytometric analyses of JC-1 staining demonstrated that inhibiting mitochondrial fission relieved the loss of mitochondrial membrane potential (MMP), which was reflected by a decreased ratio of JC-1 monomers (Supplementary Fig. 3a, b). Moreover, as shown in Supplementary Fig. 3c–f, inhibiting mitochondrial fission mitigated the increase in the production of mtROS and cellular ROS. In addition, the depletion of ATP caused by compression could also be diminished by Mdivi-1 treatment (Supplementary Fig. 3g). We then used FCCP to further confirm that suppressing mitochondrial fission was responsible for diminished mitochondrial dysfunction and ROS production. As expected, FCCP treatment impaired the inhibitory effects of Mdivi-1 on the compression-induced loss of MMP, the increase in the production of mtROS and cellular ROS, and the depletion of ATP (Supplementary Fig. 3h, i). Subsequently, we examined the expression of mitochondrial apoptotic pathway-related molecules. We found that inhibiting mitochondrial fission downregulated the expression of the proapoptotic proteins cleaved caspase 3, cleaved caspase 9 and Bax and promoted the expression of the antiapoptotic protein Bcl-2 (Supplementary Fig. 3j). Moreover, activating mitochondrial fission with FCCP counteracted these effects (Supplementary Fig. 3k). Taken together, these results indicated that compression-induced mitochondrial fission triggered apoptosis of NP cells by activating the mitochondrial apoptotic pathway.

### Promoting the expression of HSP70 attenuated compression-induced NP cell death

We first measured the expression of HSP70 in human NP tissues. As showed by IHC staining, the expression of HSP70 was elevated in degenerated human NP tissues (Supplementary Fig. 4a). We then examined the expression of HSP70 in NP cells. The PCR results showed that the expression of HSPA1A and HSPA1B was upregulated in response to compression (Supplementary Fig. 4b, c). Consistently, the protein expression of HSP70 was also increased under compression (Supplementary Fig. 4d).

**Fig. 1:**
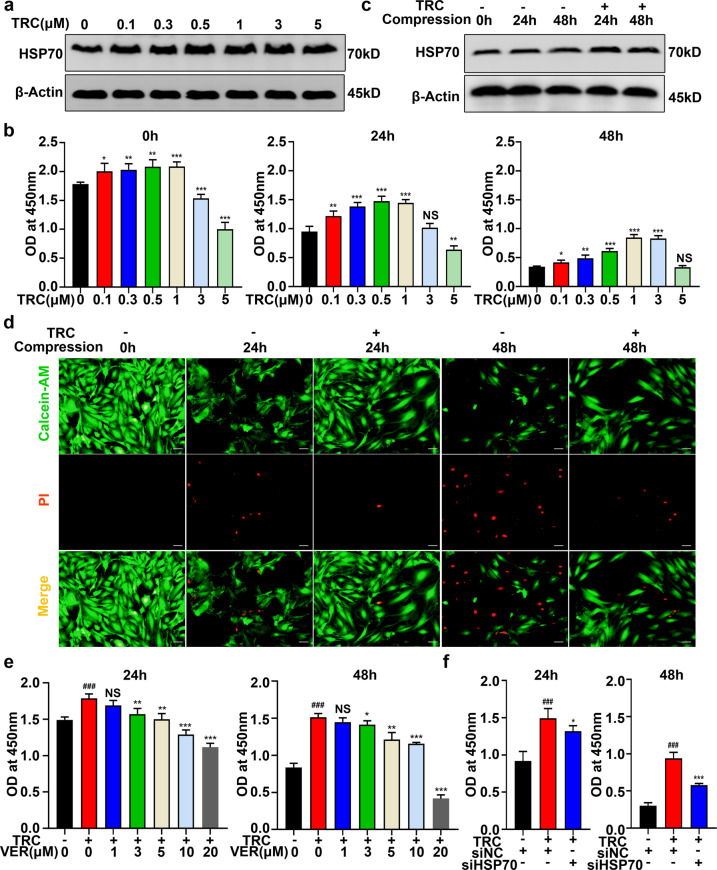
Promoting the expression of HSP70 attenuated compression-induced NP cell death. **a** The effects of different concentrations of TRC on the expression of HSP70 in NP cells (*N* = 3). **b** The effects of TRC on the viability of NP cells exposed to 0, 24, and 48 h of compression (*N* ≥ 3). **c** The effects of TRC on the expression of HSP70 in NP cells under compression (*N* = 3). **d** Representative fluorescence photomicrograph of live and dead cell staining of NP cells (*N* = 3, scale bar: 100 μm). **e** The effects of VER on the viability of NP cells treated with TRC (*N* ≥ 3). **f** The effects of HSP70 siRNA on the viability of NP cells treated with TRC (*N* ≥ 3). (*P < 0.05; **P < 0.01; ***P < 0.001 vs. the TRC 0 μM group or the TRC+, VER 0 μM group, ^###^*P* < 0.001 vs. the TRC−, VER 0 μM group or the TRC−, siNC+, siHSP70− group, NS not significant).

To investigate the role of HSP70 in IVDD, we used TRC, a novel HSP70 inducer, to promote the expression of HSP70 in NP cells^36,37^. As expected, treating NP cells with TRC for 24 h efficiently promoted the expression of HSP70 (Fig. 1a). Then, we examined the effect of TRC on the viability of NP cells. The CCK-8 assays showed that TRC could relieve the loss of viability in NP cells under compression (Fig. 1b). However, a higher concentration of TRC was toxic to NP cells (Fig. 1b). Moreover, we found that TRC (1 μM) could enhance the expression of HSP70 in NP cells under compression (Fig. 1c). We further examined the cytoprotective effects of TRC using live and dead cell staining. Correspondingly, TRC treatment decreased the ratio of dead cells and increased the ratio of live cells (Fig. 1d). To verify that the cytoprotective effects of TRC were due to increased expression of HSP70, the HSP70 inhibitor VER was used. As indicated by the CCK-8 assays, VER treatment impaired the protective effects of TRC on the loss of viability in NP cells (Fig. 1e). We further used HSP70-specific siRNA to confirm our conclusion. Following the transfection of HSP70 siRNAs, the expression of HSP70 was markedly downregulated (Supplementary Fig. 5). Furthermore, downregulating the expression of HSP70 also weakened the cytoprotective effects of TRC (Fig. 1f).

### HSP70 suppressed compression-induced apoptosis and catabolism of the extracellular matrix in NP cells

**Fig. 2:**
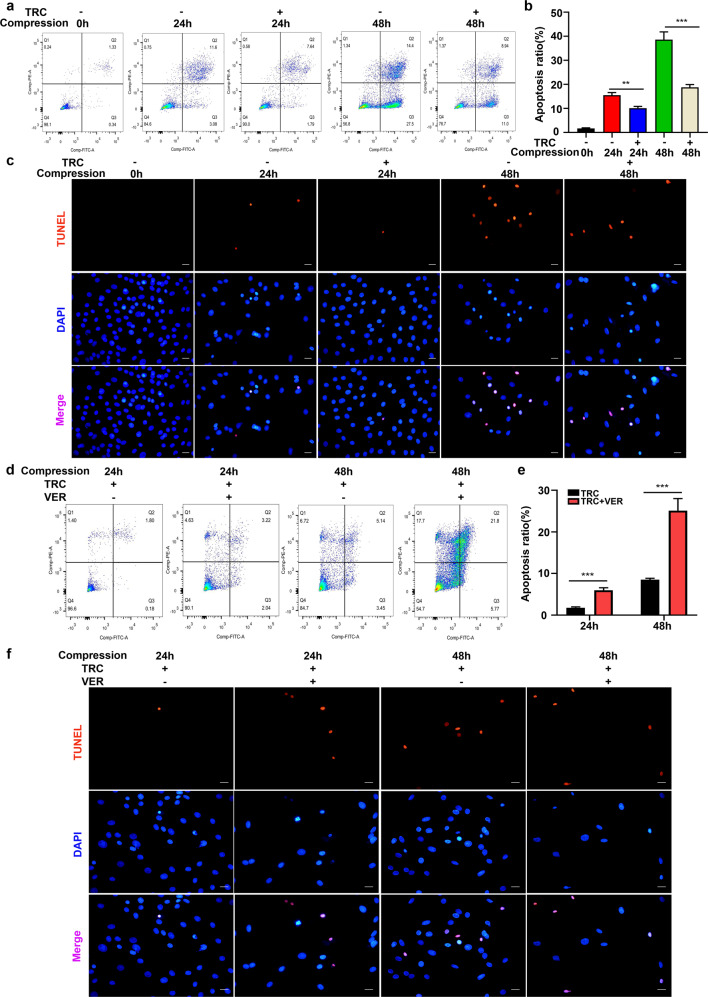
HSP70 suppressed compression-induced apoptosis of NP cells. **a**, **b** The effects of TRC on apoptosis of NP cells measured by flow cytometry using Annexin V-FITC/PI staining. **c** Typical fluorescence photomicrograph of TUNEL staining of NP cells (scale bar: 50 μm). **d**, **e** The effects of VER on apoptosis of NP cells measured by flow cytometry using Annexin V-FITC/PI staining. **f** Typical fluorescence photomicrograph of TUNEL staining of NP cells (scale bar: 50 μm). (*N* = 3, **P < 0.01; ***P < 0.001).

We then examined the role of HSP70 in compression-induced apoptosis of NP cells. Flow cytometry analyses indicated that TRC treatment efficiently alleviated apoptosis of NP cells induced by compression (Fig. 2a, b). TUNEL assay staining further verified that compression-induced apoptosis of NP cells could be suppressed by TRC (Fig. 2c). Likewise, inhibiting HSP70 with VER (30 μM) suppressed the protective effects of TRC on compression-induced apoptosis of NP cells (Fig. 2d–f). The effects of TRC on the metabolism of the extracellular matrix were also examined. We found that TRC attenuated the decrease in the expression of collagen II and aggrecan (Supplementary Fig. 6a). In addition, the upregulated expression of matrix metalloproteinases 3 and 13 was inhibited (Supplementary Fig. 6b).

### Promoting the expression of HSP70 attenuated IVDD in vivo

**Fig. 3:**
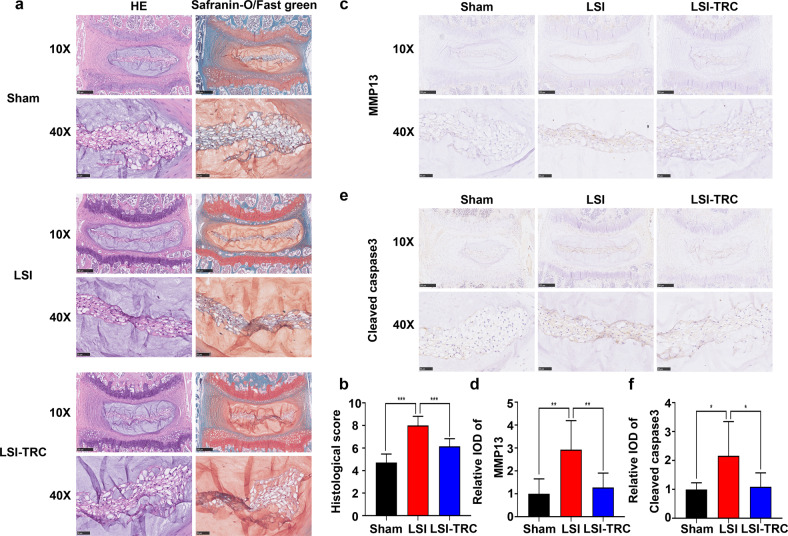
Promoting the expression of HSP70 attenuated IVDD in vivo. **a** HE and safranin-O/fast green staining of IVD samples. **b** Evaluation of IVDD by histological score. **c**, **d** IHC staining of matrix metalloproteinase 13 (MMP13) and quantitative analysis. **e**, **f** IHC staining of cleaved caspase 3 and quantitative analysis. (*n* = 7/group, *P < 0.05; **P < 0.01; ***P < 0.001).

To mimic IVDD induced by mechanical loading, the LSI model was used in the present study. As shown in Fig. 3a, compared to those in the sham group, the proportion of NP cells, the number and size of vacuoles in NP cells were decreased in the LSI group, which indicated degeneration of the IVD. In addition, TRC treatment partially ameliorated these degenerative signs of IVD. Furthermore, we evaluated the degeneration of IVDs using a grading scale as described previously^38^. The histological score also verified that TRC treatment alleviated LSI-induced degeneration of the IVD (Fig. 3b). We further measured the expression of matrix metalloproteinase 13. Consistent with the in vitro results, the expression of matrix metalloproteinase 13 increased in the LSI group, while TRC treatment decreased its expression (Fig. 3c, d). In addition, we also examined apoptosis of NP cells using IHC staining of cleaved caspase 3. We found that the expression of cleaved caspase 3 was elevated in the LSI group, while TRC treatment reduced its expression (Fig. 3e, f). Collectively, these results suggested that TRC treatment could slow the progression of IVDD in vivo by inhibiting apoptosis of NP cells.

### HSP70 inhibited the mitochondrial apoptotic pathway

**Fig. 4:**
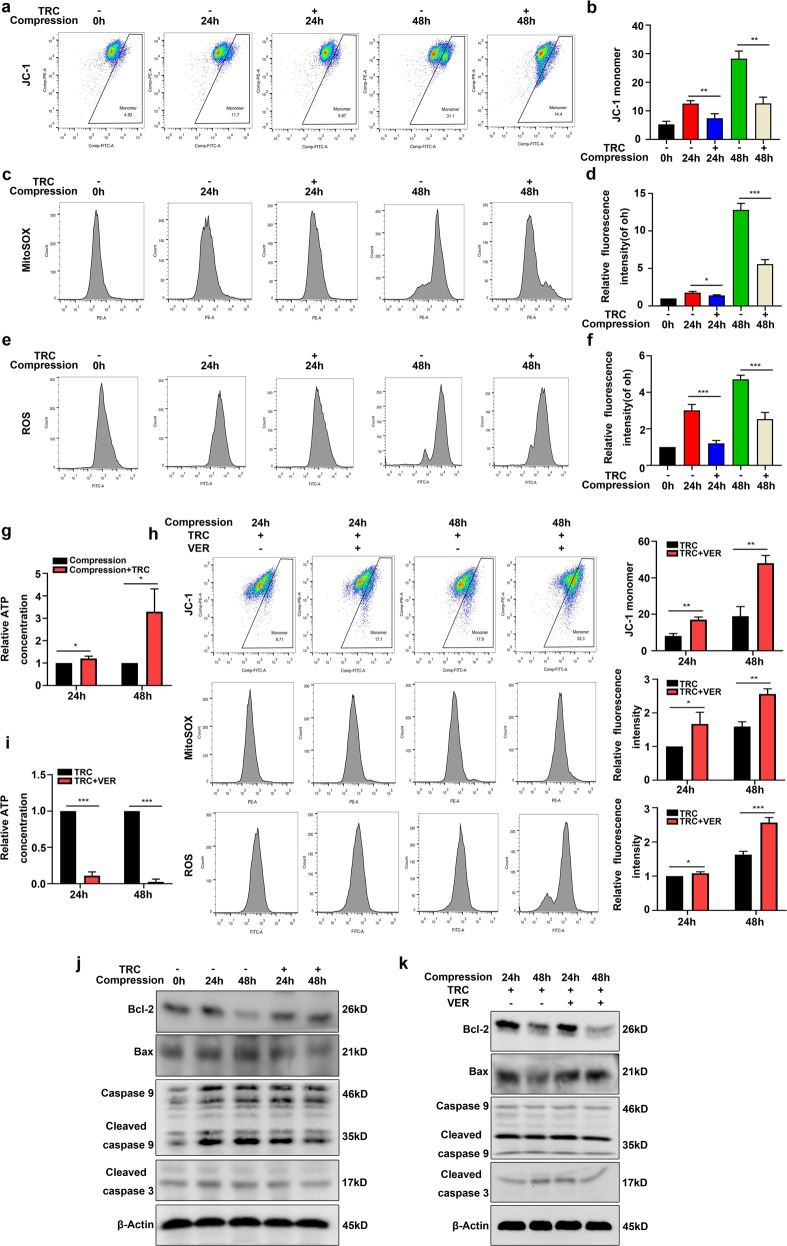
HSP70 inhibited the mitochondrial apoptotic pathway. **a**, **b** The MMP of NP cells measured by flow cytometry using JC-1 staining. **c**, **d** The production of mtROS in NP cells measured by flow cytometry using MitoSOX Red staining. **e**, **f** The production of cellular ROS in NP cells measured by flow cytometry using DCFH-DA staining. **g** The effects of TRC on ATP production in NP cells. **h** The MMP and production of mtROS and cellular ROS in NP cells measured by flow cytometry. **i** The effects of VER on ATP production in NP cells treated with TRC. **j** The effects of TRC on the expression of cleaved caspase 3, cleaved caspase 9, Bax, and Bcl-2. **k** The effects of VER on the expression of cleaved caspase 3, cleaved caspase 9, Bax, and Bcl-2 in NP cells treated with TRC. (*N* = 3, *P < 0.05; **P < 0.01; ***P < 0.001).

We then determined whether HSP70 inhibited apoptosis by blocking the mitochondrial apoptotic pathway. First, flow cytometric analyses showed that TRC treatment mitigated the loss of MMP (Fig. 4a, b). Moreover, a compression-induced increase in the production of mtROS and cellular ROS and the depletion of ATP were also inhibited by TRC treatment (Fig. 4c–g). To further confirm the involvement of HSP70 in these beneficial effects, an HSP70 inhibitor was used. We found that inhibiting HSP70 with VER impaired the protective effects of TRC on the compression-induced loss of MMP, the increased production of mtROS and cellular ROS, and the depletion of ATP (Fig. 4h, i). Finally, we evaluated the expression of mitochondrial apoptotic pathway-related molecules. The Western blot results demonstrated that TRC treatment downregulated the expression of cleaved caspase 3, cleaved caspase 9 and Bax and augmented the expression of Bcl-2 (Fig. 4j). However, VER administration neutralized these beneficial effects of TRC (Fig. 4k). Taken together, these results indicated that HSP70 suppressed compression-induced apoptosis of NP cells by blocking the mitochondrial apoptotic pathway.

### HSP70 suppressed compression-induced mitochondrial fission in NP cells

**Fig. 5:**
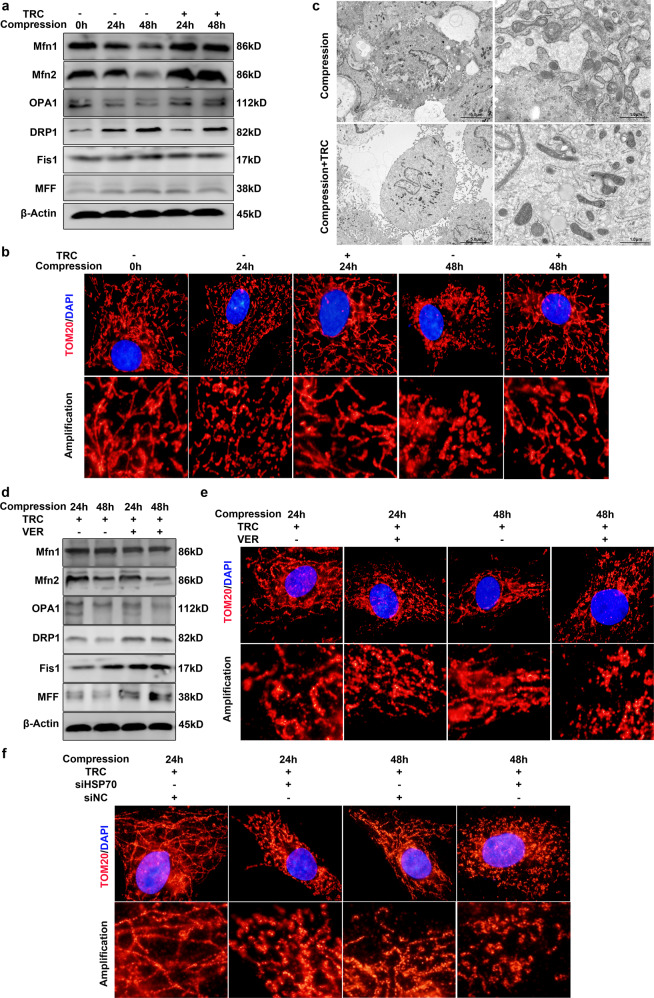
HSP70 suppressed compression-induced mitochondrial fission in NP cells. **a** The effects of TRC on the expression of DRP1, MFF, Fis1, Mfn1, Mfn2, and OPA1 (*N* = 3). **b** Representative fluorescence photomicrograph of TOM20 examined by immunofluorescence staining (*N* = 3, original magnification: ×1000). **c** The morphological ultrastructural appearance of mitochondria observed by TEM (*N* = 3). **d** The effects of VER on the expression of DRP1, MFF, Fis1, Mfn1, Mfn2, and OPA1 in NP cells treated with TRC (*N* = 3). **e** Representative fluorescence photomicrograph of TOM20 examined by immunofluorescence staining of NP cells treated with TRC and VER (*N* = 3, original magnification: ×1000). **f** Representative fluorescence photomicrograph of TOM20 examined by immunofluorescence staining of NP cells treated with TRC and HSP70-specific siRNAs (*N* = 3, original magnification: ×1000).

To explain how HSP70 inhibited the mitochondrial apoptotic pathway, we focused on the role of HSP70 in mitochondrial fission and fusion. The Western blot results demonstrated that TRC treatment downregulated the expression of DRP1, Fis1 and MFF while enhancing the expression of Mfn1, Mfn2, and OPA1 (Fig. 5a). Moreover, IF staining showed that mitochondrial fission caused by compression was alleviated by TRC administration (Fig. 5b). Furthermore, TEM also demonstrated that TRC administration inhibited the fragmentation of mitochondria in NP cells (Fig. 5c). Next, we used an HSP70 inhibitor to further confirm the inhibitory effects of HSP70 on mitochondrial fission. Consistently, inhibiting HSP70 with VER increased the expression of DRP1, Fis1 and MFF while decreasing the expression of Mfn1, Mfn2, and OPA1 (Fig. 5d). Moreover, VER also impaired the inhibitory effects of TRC on the fragmentation of mitochondria in response to compression (Fig. 5e). Furthermore, downregulating the expression of HSP70 also weakened the inhibitory effects of TRC on mitochondrial fission in NP cells (Fig. 5f). Collectively, these evidence suggests that HSP70 suppresses compression-induced mitochondrial fission in NP cells.

### HSP70 inhibited the mitochondrial apoptotic pathway by suppressing mitochondrial fission

**Fig. 6:**
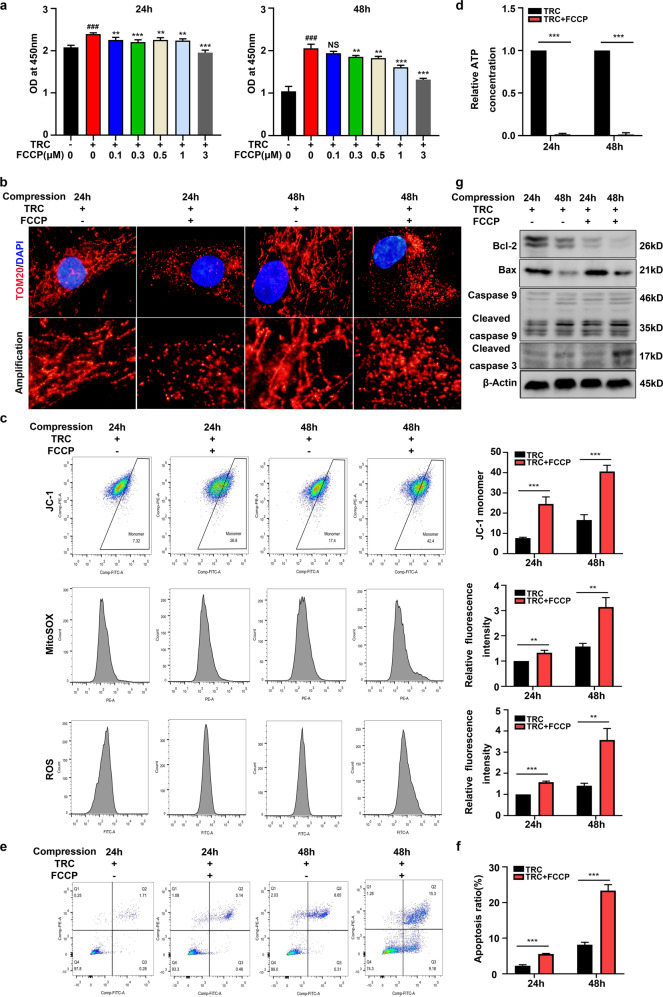
HSP70 inhibited the mitochondrial apoptotic pathway by suppressing mitochondrial fission. **a** The effects of FCCP on the viability of NP cells treated with TRC (*N* ≥ 3). **b** Representative fluorescence photomicrograph of TOM20 examined by immunofluorescence staining of NP cells treated with TRC and FCCP (*N* = 3, original magnification: ×1000). **c** The MMP and the production of mtROS and cellular ROS in NP cells were measured by flow cytometry (*N* = 3). **d** The effects of FCCP on ATP production in NP cells treated with TRC (*N* = 3). **e**, **f** The effects of FCCP on apoptosis of NP cells measured by flow cytometry using Annexin V-FITC/PI staining (*N* = 3). **g** The effects of FCCP on the expression of cleaved caspase 3, cleaved caspase 9, Bax, and Bcl-2 in NP cells treated with TRC (*N* = 3). (**P < 0.01; ***P < 0.001 vs. the control group or the TRC+, FCCP 0 μM group, ^###^*P* < 0.001 vs. the TRC−, FCCP 0 μM group, NS not significant).

We subsequently explored whether HSP70 inhibited the mitochondrial apoptotic pathway by suppressing mitochondrial fission. The CCK-8 assay results showed that FCCP treatment impaired the protective effects of TRC on the loss of viability in NP cells (Fig. 6a). IF staining of TOM20 showed that FCCP (3 μM) markedly recall mitochondrial fission in NP cells treated with TRC (Fig. 6b). Following the reactivation of mitochondrial fission, the protective effects of TRC on the compression-induced loss of MMP, the increased production of mtROS and cellular ROS, and the depletion of ATP also disappeared (Fig. 6c, d). Furthermore, FCCP treatment also counteracted the protective effects of TRC on compression-induced apoptosis of NP cells (Fig. 6e, f). Consistently, TRC-induced downregulation of cleaved caspase 3, cleaved caspase 9 and Bax and the upregulation of Bcl-2 were also abolished by FCCP treatment (Fig. 6g).

### HSP70 suppressed compression-induced mitochondrial fission by upregulating SIRT3 expression

**Fig. 7:**
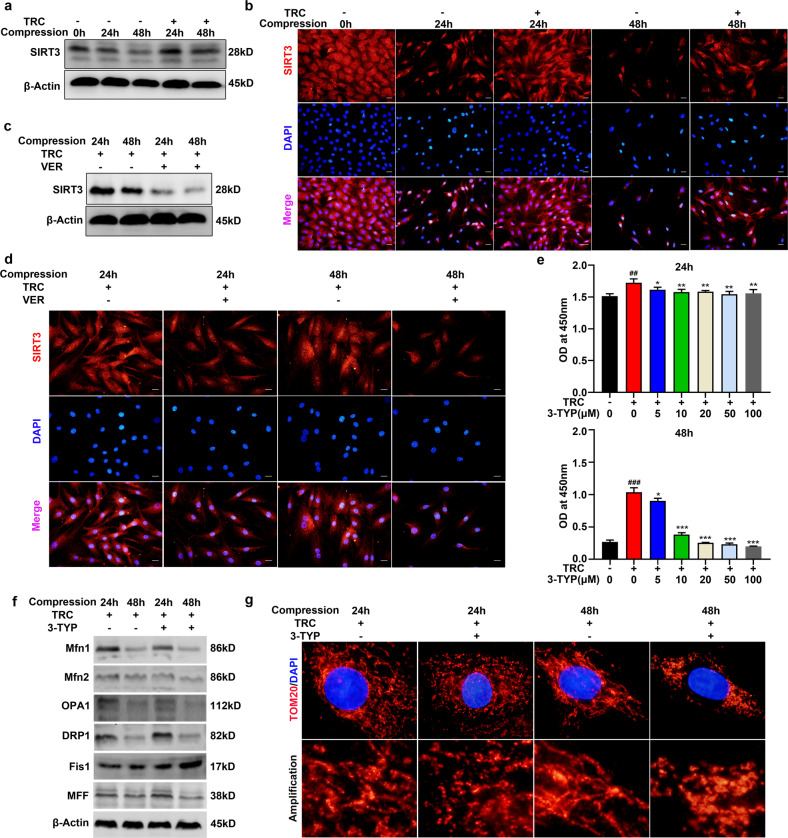
HSP70 suppressed compression-induced mitochondrial fission by upregulating SIRT3 expression. **a** The effects of TRC on SIRT3 expression (*N* = 3). **b** Representative fluorescence photomicrograph of SIRT3 expression examined by immunofluorescence staining (*N* = 3, scale bar: 50 μm). **c** The effects of VER on SIRT3 expression in NP cells treated with TRC (*N* = 3). **d** Representative fluorescence photomicrograph of SIRT3 expression examined by immunofluorescence staining (*N* = 3, scale bar: 50 μm). **e** The effects of 3-TYP on the viability of NP cells treated with TRC (*N* ≥ 3). **f** The effects of 3-TYP on the expression of DRP1, MFF, Fis1, Mfn1, Mfn2, and OPA1 in NP cells treated with TRC (*N* = 3). **g** Representative fluorescence photomicrograph of TOM20 examined by immunofluorescence staining of NP cells treated with TRC and 3-TYP (*N* = 3, original magnification: ×1000). (*P < 0.05; **P < 0.01; ***P < 0.001 vs. the TRC+, 3-TYP 0 μM group, ^##^*P* < 0.01, ^###^*P* < 0.001 vs. the TRC−, 3-TYP 0 μM group).

To understand how HSP70 suppressed compression-induced mitochondrial fission, we focused on the regulatory effects of HSP70 on SIRT3. We first measured SIRT3 expression in human NP tissues. IHC staining showed that SIRT3 expression was downregulated in degenerated human NP tissues (Supplementary Fig. 7). Then, we examined the effect of TRC treatment on SIRT3 expression. The Western blot results demonstrated that TRC treatment augmented SIRT3 expression (Fig. 7a). IF staining further verified the upregulation of SIRT3 induced by TRC treatment (Fig. 7b). In contrast, following the administration of VER, the upregulation of SIRT3 protein expression was inhibited (Fig. 7c). IF staining also revealed the same trend (Fig. 7d). We subsequently determined the effects of upregulated SIRT3 expression on mitochondrial fission and fusion using the SIRT3 inhibitor 3-TYP. We found that 3-TYP could impair the protective effects of TRC on the compression-induced loss of viability of NP cells (Fig. 7e). Moreover, 3-TYP (50 μM) treatment increased the expression of DRP1, Fis1, and MFF and decreased the expression of Mfn1, Mfn2, and OPA1 (Fig. 7f). IF staining of TOM20 further showed that 3-TYP impaired the inhibitory effects of TRC on compression-induced mitochondrial fission in NP cells (Fig. 7g).

### SIRT3 mediated the inhibitory effects of HSP70 on the mitochondrial apoptotic pathway

**Fig. 8:**
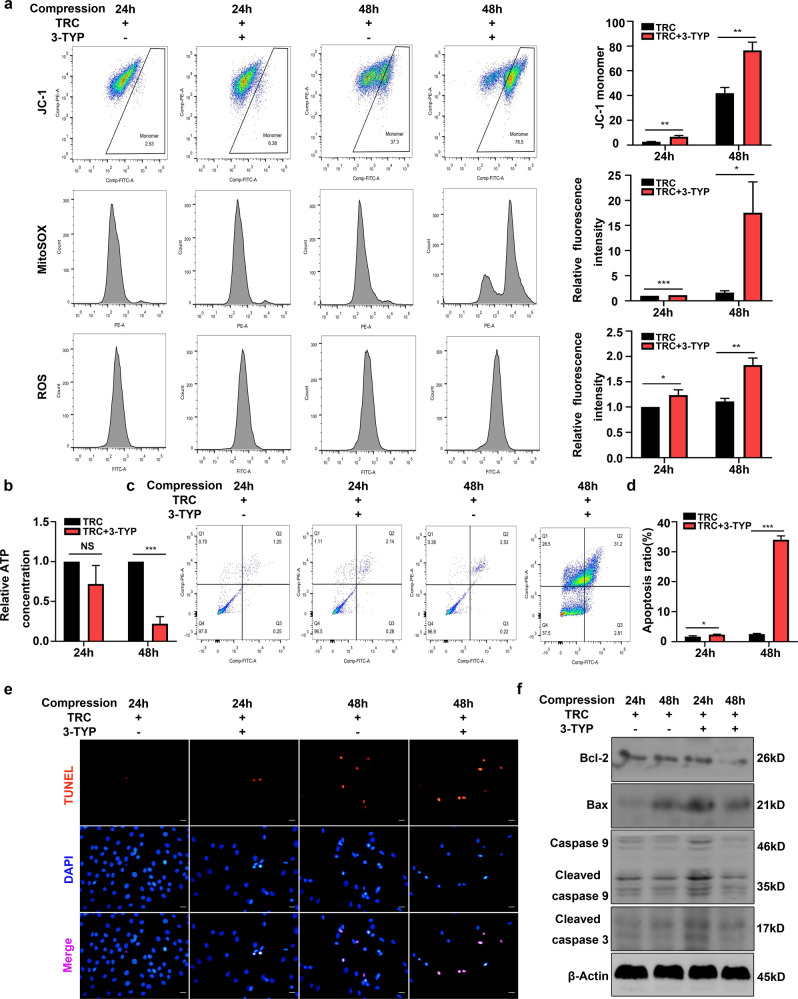
SIRT3 mediated the inhibitory effects of HSP70 on the mitochondrial apoptotic pathway. **a** The MMP and the production of mtROS and cellular ROS in NP cells measured by flow cytometry. **b** The effects of 3-TYP on ATP production in NP cells treated with TRC. **c**, **d** The effects of 3-TYP on apoptosis of NP cells measured by flow cytometry using Annexin V-FITC/PI staining. **e** Typical fluorescence photomicrograph of TUNEL staining in NP cells (scale bar: 50 μm). **f** The effects of 3-TYP on the expression of cleaved caspase 3, cleaved caspase 9, Bax, and Bcl-2 in NP cells treated with TRC. (*N* = 3, *P < 0.05, **P < 0.01; ***P < 0.001, NS not significant).

We then examined whether upregulated SIRT3 expression was responsible for the inhibitory effects of HSP70 on the mitochondrial apoptotic pathway. As expected, flow cytometric analyses showed that following 3-TYP treatment, the preventive effects of TRC on the compression-induced loss of MMP, the increased production of mtROS and cellular ROS, and the depletion of ATP disappeared (Fig. 8a, b). Moreover, flow cytometric analyses indicated that 3-TYP treatment recalled apoptosis of NP cells treated with TRC (Fig. 8c, d). Consistently, TUNEL staining also revealed the same trend (Fig. 8e). Furthermore, the TRC-induced downregulation of cleaved caspase 3, cleaved caspase 9 and Bax and upregulation of Bcl-2 were abolished by 3-TYP treatment (Fig. 8f). Taken together, these results revealed that upregulated expression of SIRT3 mediated the inhibitory effects of HSP70 on the mitochondrial apoptotic pathway.

## Discussion

Compression-induced apoptosis of NP cells plays pivotal roles in the pathogenesis of IVDD; however, its mechanisms and regulatory effects have not yet been fully elucidated. In the present study, we found that compression could induce mitochondrial fission, which ultimately triggered apoptosis of NP cells via the mitochondrial apoptotic pathway. In addition, we identified the cytoprotective effects of HSP70 on NP cells, and we found that promoting the expression of HSP70 could protect NP cells from abnormal mechanical loading in vitro and in vivo. Finally, we proved that HSP70 inhibited compression-induced mitochondrial fission by promoting SIRT3 expression, thereby attenuating mitochondrial dysfunction and the production of ROS and ultimately inhibiting the mitochondrial apoptotic pathway in NP cells. To the best of our knowledge, this is the first study to uncover the regulatory effects of HSP70 on SIRT3 expression and downstream mitochondrial fission and fusion.

The dysregulation of mitochondrial fission and fusion has been implicated in the pathogenesis and progression of a variety of diseases^11,12^. In IVDD, mitochondria are fragmented in aged NP cells, indicating the potential involvement of mitochondrial fission in IVDD^39^. Moreover, many in vitro studies further revealed that adverse stimuli could trigger mitochondrial fission in IVD cells^21,40,41^. Accordingly, preventing mitochondrial fission was proven to be cytoprotective in IVD cells^42^. In the present study, we found that the expression of DRP1 and MFF increased while the expression of Mfn1 and Mfn2 decreased in human degenerated NP tissues, which further provided in vivo evidence for the involvement of mitochondrial fission in the pathogenesis of IVDD. Abnormal mechanical loading is the leading cause of IVDD. A previous study demonstrated that compression augmented the expression of mitochondrial fission-related proteins and downregulated the expression of mitochondrial fusion-related proteins in NP cells^14^. However, because proteins involved in the regulation of mitochondrial fission and fusion might also mediate other physiological processes, there is still a lack of direct evidence to prove the causal relationship between compression and mitochondrial fission in NP cells^43,44^. Herein, we used IF staining of TOM20 and TEM to directly observe mitochondrial morphology. Combined with measuring the expression of mitochondrial fission- and fusion-related proteins, our results proved that compression triggered mitochondrial fission in NP cells.

Excessive mitochondrial fission is closely associated with mitochondrial dysfunction. It was demonstrated that during the process of heart ischemia–reperfusion, excessive mitochondrial fission could cause the uneven division of mitochondrial DNA, thus disturbing the replication and transcription of mtDNA^45,46^. Moreover, mitochondrial fission could impair the activities of respiratory chain complexes^19^. Using an inhibitor and activator of mitochondrial fission, we found that compression-induced mitochondrial fission caused mitochondrial dysfunction in NP cells. Considering the important role of mitochondrial fission in mitochondrial dysfunction, an increasing amount of evidence has confirmed the proapoptotic role of mitochondrial fission via the activation of the mitochondrial apoptotic pathway^8^. Excessive mitochondrial fission could generate enormous amounts of mitochondrial debris containing lower MMP and fragmentary DNA, which release proapoptotic factors into the cytoplasm and nucleus and triggers the mitochondrial apoptotic pathway^18,47,48^. Moreover, mitochondrial fission could initiate apoptosis by promoting calcium overload in mitochondria^49^. Accordingly, inhibiting mitochondrial fission has been proven to suppress apoptosis triggered by various adverse stimuli^50^. In the current study, we not only proved that inhibiting mitochondrial fission attenuated compression-induced apoptosis but also revealed that reactivating mitochondrial fission recalled apoptosis in NP cells treated with Mdivi-1. Thus, our results verified that compression-induced mitochondrial fission was responsible for apoptosis of NP cells.

HSP70 is the most important cytoprotective heat shock protein, and its expression is largely induced when cells are exposed to unfavorable stimuli^51^. NP cells are continuously subjected to insults due to adverse microenvironmental factors within the IVD. A previous study showed that the expression of HSP70 was increased in degenerated IVDs^52^. Moreover, several in vitro studies verified that hypoxia, hypertonia, and mechanical loading upregulated the expression of HSP70 in NP cells^26–28^. Consistently, we identified increased expression of HSP70 in degenerated NP tissues and compression-treated NP cells. These results indicated that in response to adverse stimuli, NP cells could initiate a resistance mechanism to induce the expression of HSP70. However, although the expression of HSP70 is increased, IVDD still inevitably occurs. We hypothesized that several factors may be involved in the failure of the cytoprotective effects of HSP70. First, accompanied by increased expression of HSP70, the expression of HSP90 was elevated in degenerated NP tissues, and compression could promote the expression of HSP90 in NP cells^28,34^. However, it was proven that HSP90 suppressed the expression of HSP70^53^. Furthermore, NP cells are lastingly exposed to various adverse microenvironmental factors, which might impair the cytoprotective effects of HSP70. Due to these factors, the increase in the expression of HSP70 was insufficient to protect NP cells from insults of various adverse stimuli, and the occurrence of IVDD was therefore inevitable.

We further explored whether the induction of HSP70 attenuated the progression of IVDD. Accumulating evidence has shown that the induction of HSP70 effectively prevents the development of various diseases. It was shown that promoting the expression of HSP70 inhibited calcium overload in myocytes, thereby alleviating ischemia–reperfusion injury^54^. The induction of HSP70 could protect the brain and brain cells from experimental cerebral ischemia, neurodegenerative disease, epilepsy, and trauma^23^. Accordingly, we revealed that promoting the expression of HSP70 prevented compression-induced death and degradation of the extracellular matrix in NP cells. HSP70 could regulate the mitochondrial apoptotic pathway at different levels. Consistently, we found that HSP70 prevented compression-induced mitochondrial dysfunction and ROS production in NP cells, ultimately inhibiting the activation of the mitochondrial apoptotic pathway. Furthermore, in vivo experiments verified that the induction of HSP70 slowed the progression of IVDD. Thus, our results indicated that promoting the expression of HSP70 might be a novel strategy for the treatment of IVDD.

The above results suggested that both the inhibition of mitochondrial fission and the induction of HSP70 prevented compression-induced apoptosis of NP cells. However, whether HSP70 inhibits apoptosis by suppressing mitochondrial fission has not yet been determined. A previous study showed that the loss of mitochondrial HSP70 function makes the tubular network of mitochondria collapse to form aggregates in yeast cells, which indicated that HSP70 might have the capacity to regulate mitochondrial fission and fusion^29^. Interestingly, by measuring the expression of mitochondrial fission- and fusion-related molecules and observing mitochondrial morphology directly by IF staining and TEM, we found that HSP70 could effectively inhibit mitochondrial fission in NP cells. Furthermore, we identified that the suppression of mitochondrial fission was responsible for the inhibitory effects of HSP70 on compression-induced mitochondrial dysfunction and the activation of the mitochondrial apoptotic pathway.

To elucidate how HSP70 suppressed compression-induced mitochondrial fission, we focused on the regulatory effects of HSP70 on SIRT3. Although the regulatory effects of HSP70 on SIRT3 have not been documented previously, it was proven that both SIRT1 and SIRT3 could interact with HSP70, and HSP70 has been demonstrated to regulate the expression of SIRT1^30,31^. Similarly, we found that HSP70 promoted SIRT3 expression in NP cells. We hypothesized that the promoting effects of HSP70 on SIRT3 expression might be due to its role as a molecular chaperone to prevent the degradation of SIRT3. However, further research is needed to validate our hypothesis. Previous studies have shown that SIRT3 can inhibit the expression of Fis1 and promote the expression and activation of OPA1, thus suppressing mitochondrial fission^18–20^. Correspondingly, we identified the regulatory effects of SIRT3 on the expression of Fis1 and OPA1. In addition, we established that SIRT3 could regulate the expression of Mfn1, Mfn2, DRP1, and MFF. Therefore, our results verified that HSP70 suppressed compression-induced mitochondrial fission by upregulating SIRT3 expression. Furthermore, our results demonstrated that SIRT3 mediated the inhibitory effects of HSP70 on compression-induced mitochondrial dysfunction and the activation of the mitochondrial apoptotic pathway in NP cells. However, we must note that in addition to SIRT3, there might be other mechanisms involved in the regulatory effects of HSP70 on mitochondrial fission, which need further exploration.

In conclusion, our results demonstrated that compression-induced mitochondrial fission could trigger apoptosis of NP cells by activating the mitochondrial apoptotic pathway. Furthermore, we demonstrated for the first time that promoting the expression of HSP70 could attenuate compression-induced apoptosis of NP cells by suppressing mitochondrial fission via upregulating SIRT3 expression. Our results indicated that promoting the expression of HSP70 might be a novel strategy for the treatment of IVDD.

## Supplementary information

Supplementary figures
