# Supplementary material for: HSP70 attenuates compression-induced apoptosis of nucleus pulposus cells by suppressing mitochondrial fission via upregulating the expression of SIRT3
Source: Exp Mol Med. 2022 Mar 25;54(3):309–23. doi: 10.1038/s12276-022-00745-9 (PMC8980024; doi:10.1038/s12276-022-00745-9)
Supplement: Supplementary file 1 — Supplementary figures [file 12276_2022_745_MOESM1_ESM.pdf]

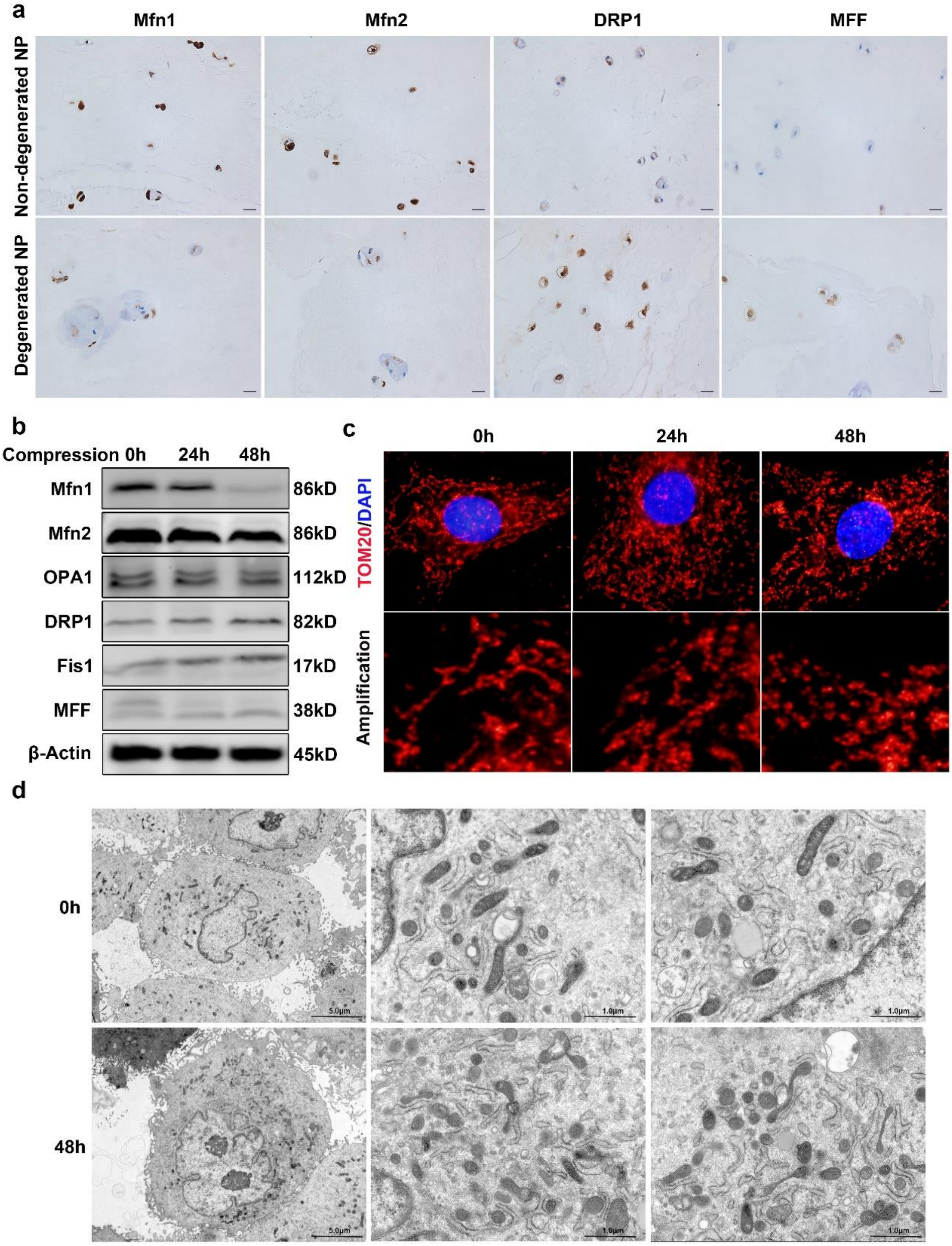

Supplementary Fig. 1 Compression induced the mitochondrial fission of NP cells. (a) IHC staining of Mfn1, Mfn2, DRP1 and MFF in non-degenerated and degenerated human NP tissues (n = 5/group, scale bar: 50 μm). (b) The effects of compression on the expression of DRP1, MFF, Fis1, Mfn1, Mfn2 and OPA1 (N=3). (c) The

representative fluorescence photomicrograph of TOM20 detected by immunofluorescence staining (N=3, original magnification: ×1000). (d) The morphological ultrastructural appearance of mitochondria observed by TEM (N=3).

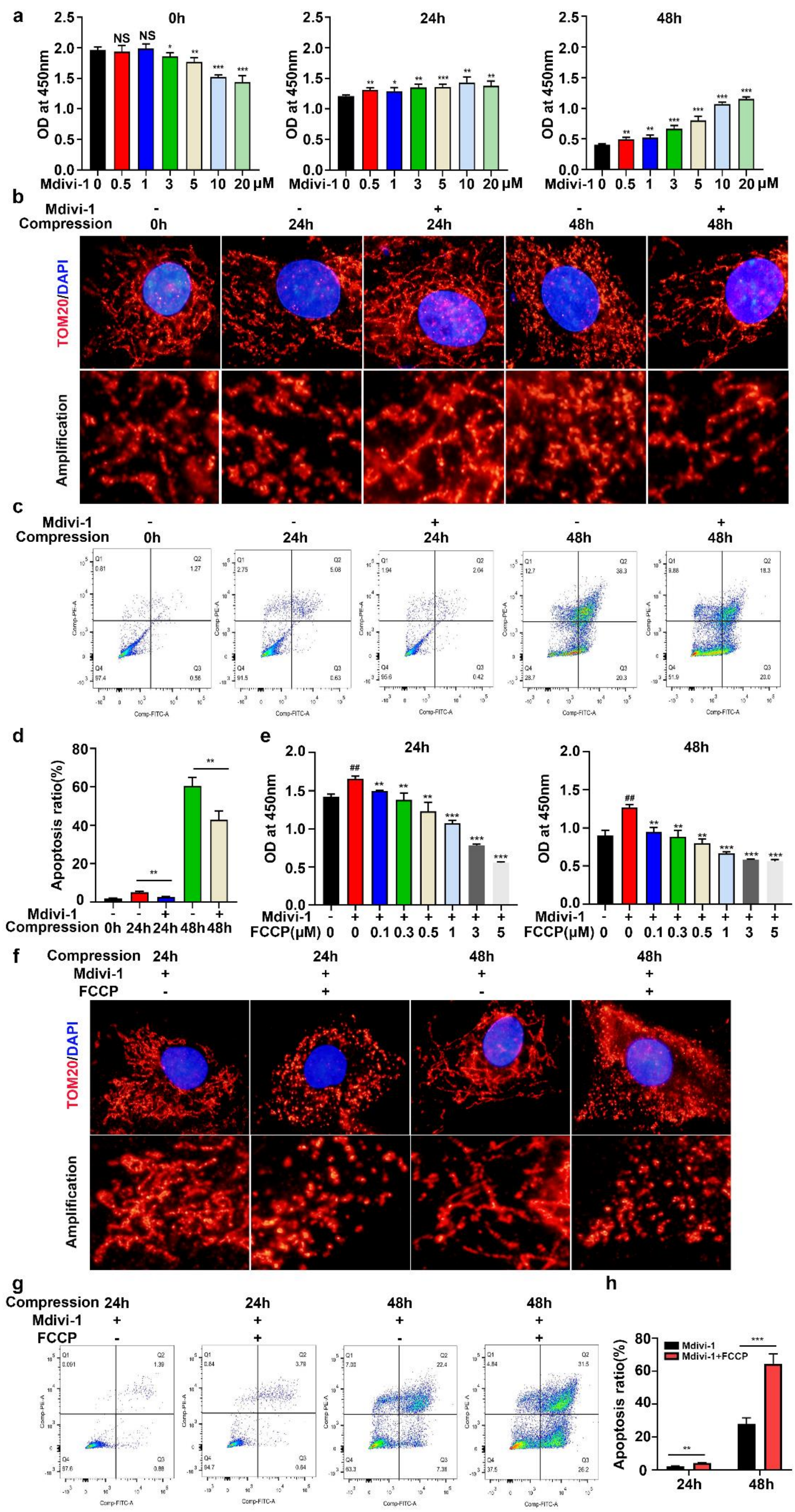
a
0h
24h
48h
OD at 450nm
Mdivi-1 0 0.5 1 3 5 10 20 μM
b
Mdivi-1
Compression
TOM20/DAPI
Amplification
c
Mdivi-1
Compression
Comp-PE-A
Comp-FITC-A
d
Apoptosis ratio(%)
e
FCCP(μM)
f
FCCP
g
h
Mdivi-1
Mdivi-1+FCCP

Supplementary Fig. 2 Inhibiting mitochondrial fission attenuated compression-induced apoptosis of NP cells. (a) The effects of Mdivi-1 on the viability of NP cells exposed to 0 h, 24 h and 48 h of compression (N⩾3). (b) The representative fluorescence photomicrograph of TOM20 detected by immunofluorescence staining (N=3, original magnification: ×1000). (c-d) The apoptosis ratio of NP cells measured by flow cytometric analyses using the Annexin V-FITC/PI staining (N=3). (e) The effects of FCCP on the viability of NP cells treated with Mdivi-1 under compression (N⩾3). (f) The representative fluorescence photomicrograph of TOM20 detected by immunofluorescence staining (N=3, original magnification: ×1000). (g-h) The apoptosis ratio of NP cells measured by flow cytometric analyses using the Annexin V-FITC/PI staining (N=3). (*P< 0.05, **P< 0.01, ***P< 0.001 vs. the control group, the Mdivi-1 0 μM group or the Mdivi-1+, FCCP 0 μM group, $^{\#\#}P$< 0.01 vs. the Mdivi-1-, FCCP 0 μM group, NS, not significant).

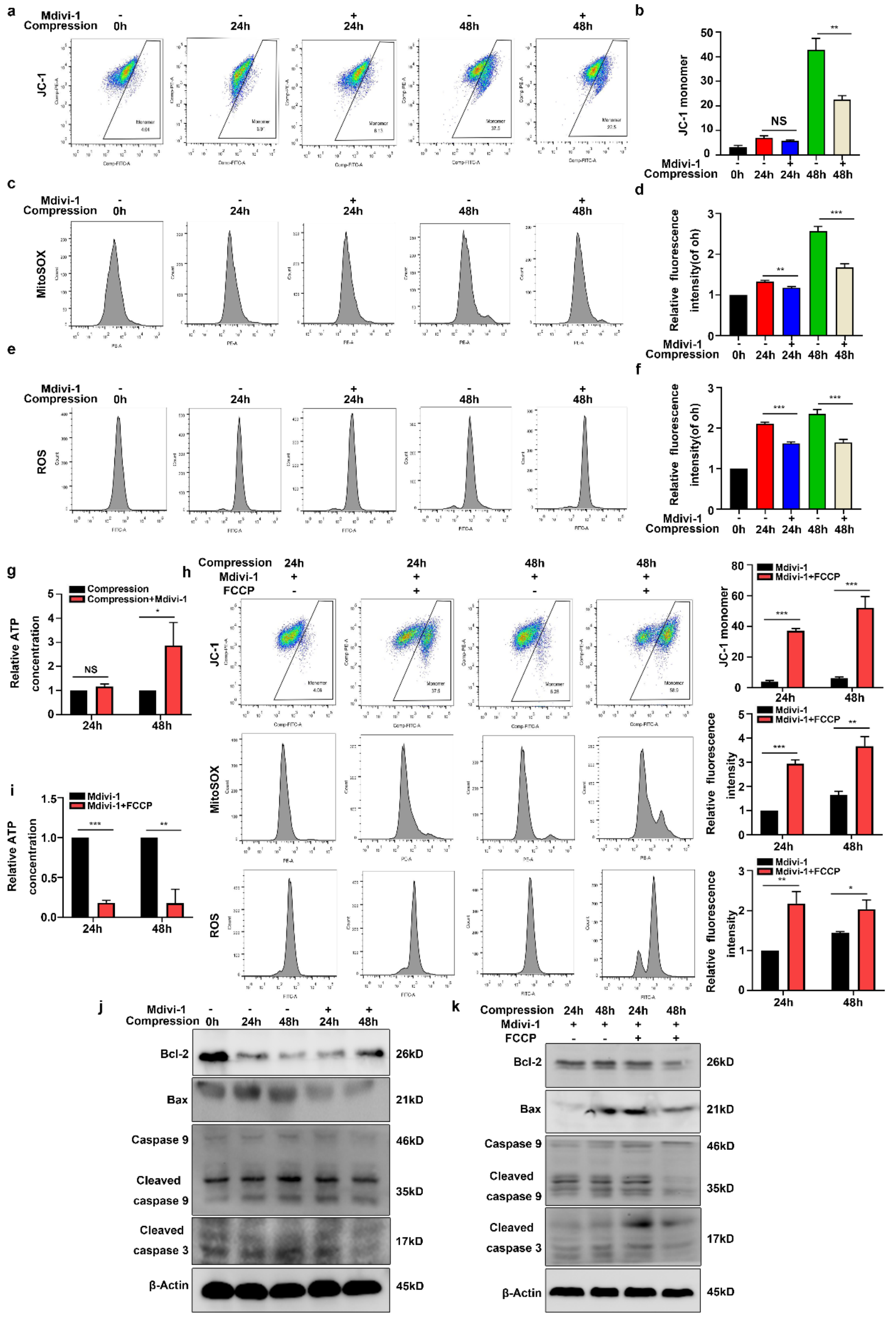

Supplementary Fig. 3 Inhibiting mitochondrial fission suppressed the mitochondrial apoptotic pathway. (a-b) The MMP of NP cells measured by flow cytometric analyses

using the JC-1 staining. (c-d) The production of mtROS in NP cells measured by flow cytometric analyses using the MitoSOX Red staining. (e-f) The production of cellular ROS in NP cells measured by flow cytometric analyses using the DCFH-DA staining. (g) The effects of Mdivi-1 on the production ATP in NP cells. (h) The MMP, production of mtROS and cellular ROS in NP cells measured by flow cytometric analyses. (i) The effects of FCCP on the production ATP in NP cells treated with FCCP. (j) The effects of Mdivi-1 on the expression of cleaved caspase 3, cleaved caspase 9, Bax and Bcl-2. (k) The effects of FCCP on the expression of cleaved caspase 3, cleaved caspase 9, Bax and Bcl-2 in NP cells treated with Mdivi-1. (N=3, *P< 0.05, **P< 0.01, ***P< 0.001, NS, not significant).

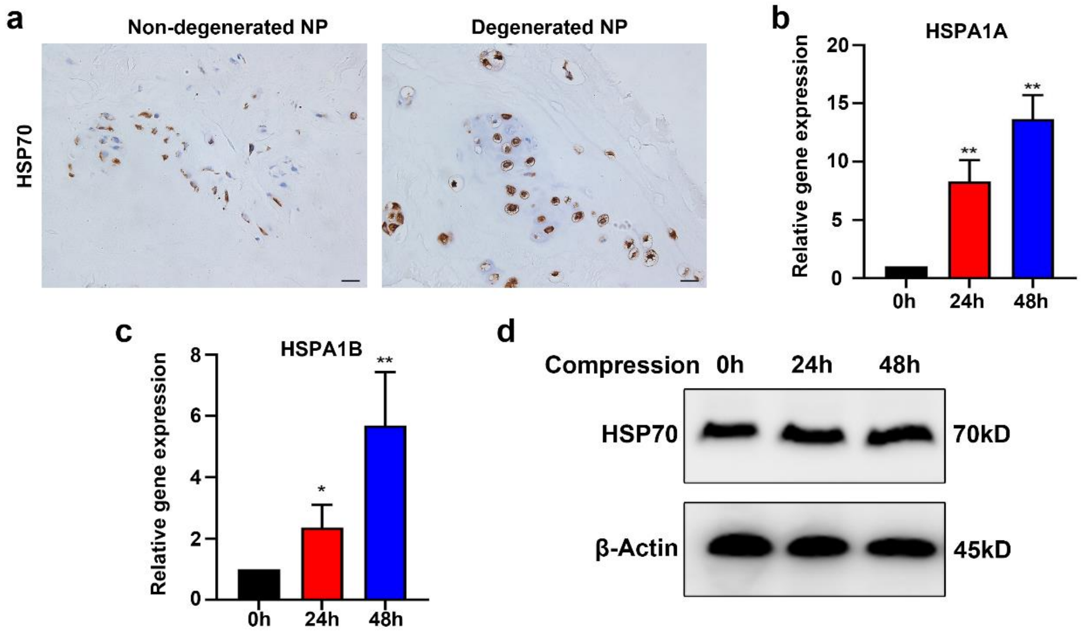

Supplementary Fig. 4 The expression of HSP70 in NP tissues and NP cells. (a) IHC staining of HSP70 in non-degenerated and degenerated human NP tissues (n = 5/group, scale bar: 50 μm). (b-c) The expression levels of HSPA1A and HSPA1B measured by RT-PCR in NP cells exposed to 0 h, 24 h and 48 h of compression (N=3). (d) The effects of compression on the expression of HSP70 (N=3). (*$P< 0.05$, **$P< 0.01$ vs. 0 h).

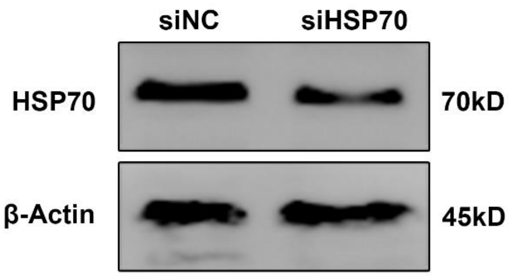

Supplementary Fig. 5 The transfection efficacy of HSP70 siRNAs evaluated by Western blot (N=3).

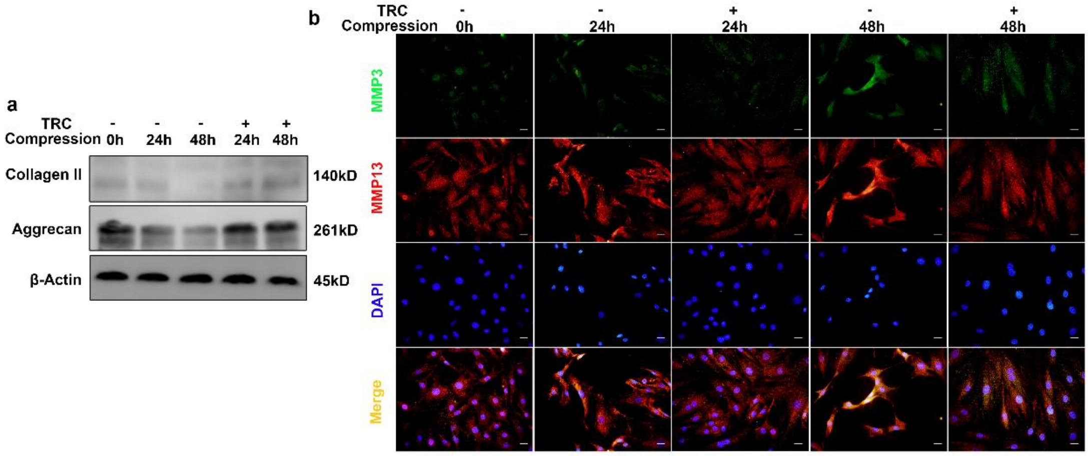

Supplementary Fig. 6 The effects of HSP70 on the metabolism of extracellular matrix in NP cells. (a) The effects of TRC on the expression of collagen II and aggrecan (N=3). (b) The representative fluorescence photomicrograph of matrix metalloproteinase 3 and 13 (MMP3 and 13) expression detected by immunofluorescence staining (N=3, scale bar: 50 μm).

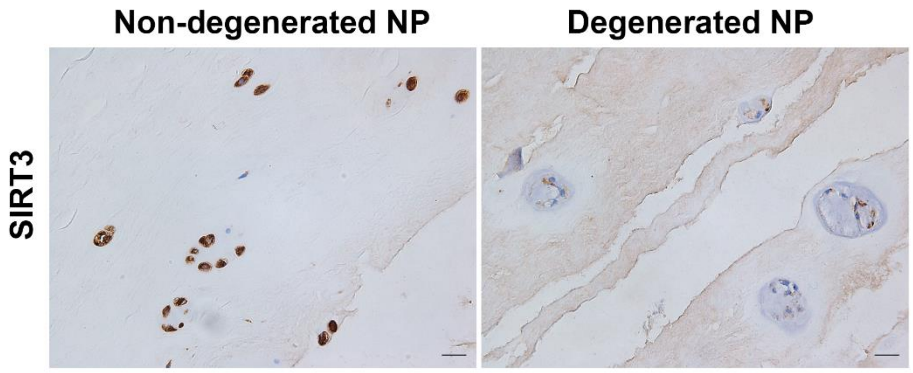

Supplementary Fig. 7 IHC staining of SIRT3 in non-degenerated and degenerated human NP tissues (n = 5/group, scale bar: 50 μm).
